# The Values of Coronary Circulating miRNAs in Patients with Atrial Fibrillation

**DOI:** 10.1371/journal.pone.0166235

**Published:** 2016-11-17

**Authors:** Guiyu Xu, Yuxia Cui, Zhenghua Jia, Yunan Yue, Shuixiang Yang

**Affiliations:** 1 The Department of Cardiology, Capital Medical University Affiliated Beijing Shijitan Hospital, Beijing, China; 2 The Dept. of Cardiology, Hebei Medical University Affiliated Yiling Hospital, Hebei, China; University of Bologna, ITALY

## Abstract

The mechanism of miRNA regulation in atrial fibrillation (AF) occurrence and development is still unclear, especially, the regulating values of coronary circulating miRNAs has not been reported. Based on our AF radiofrequency ablation clinical practice and previous miRNA study, we proposed a hypothesis that the coronary circulating miRNA might much better reflect the regulating state and metabolic level of myocardial miRNA in AF patient. To investigate the regulating values of coronary circulation miRNA, 90 AF patients were selected and compared with 90 healthy subjects, the changes of coronary circulating miRNA differential expression profile in the whole genome were observed in this study. We found out that compared with autologous peripheral blood (PB), 6 miRNAs were upregulated and 8 miRNA downregulated in AF patients’ coronary sinus blood (CSB) significantly, especially, the expression of miR-1266, miR-4279 and miR-4666a-3p were obviously increased. Compared with normal donors’ peripheral blood, 16 miRNAs were upregulated and 24 miRNAs downregulated dramatically in patients’ peripheral blood, among them, the miR-3171 decreased, but miR-892a and miR-3149 increased significantly from the early to end stages of AF. Our results indicated that the coronary circulating miRNA can really reflect the regulating values of miRNA in AF patient; the level of miRNA change in 3 types of AF may reflect the severity of AF clinical and pathophysiological advance; The miR-892a, miR-3171 and miR-3149 may be used as biomarkers for earlier diagnosis, while miR-1266, miR-4279 and miR-4666a-3p may serve as potential intervening targets for AF patient in future.

## Introduction

Despite the fact that the pathophysiology of atrial fibrillation (AF) has been investigated extensively for almost a century, the underlying mechanisms remain only partially understood [1–5]. Conventional theories have focused on electrical and structural remodeling [6]. But the mechanism of miRNA regulation in atrial fibrillation (AF) occurrence and development, especially, the effects of miRNA on cardiac remodeling is still not fully clear [7–9], furthermore, the regulating values of coronary circulating miRNA has not been reported until now.

MicroRNAs (miRNAs) are short, endogenous, noncoding RNAs that regulate gene expression at the posttranscriptional level by binding to the 3’ untranslated regions (UTRs) of their target mRNAs. miRNAs are thought to play a critical role in regulating the expression of various genes that contribute to AF [10, 11]. Recently, miRNAs detected in the circulating serum and atrial tissue have been reported [12–19]. However, those data cannot totally explain the regulation mechanisms of miRNAs in AF; also, differential expression of miRNAs still cannot be used as biomarkers in the early diagnosis of and intervention on AF at present.

Based on our AF radiofrequency ablation clinical practice and previous miRNA study [20–23], we proposed a hypothesis that the coronary circulating miRNA might much better reflect the regulating state and metabolic level of myocardial miRNA in AF patient. To investigate the regulating values of coronary circulation miRNA, 90 AF patients were selected and the coronary sinus blood (CSB) was taken from during the ablation operation, compared with 90 healthy subjects, the changes of coronary circulating miRNA differential expression profile in the whole genome were observed in this study. We found out that the coronary circulating miRNA can really reflect the regulating values of miRNA in AF patient; we also try to find out the miRNA regulating significance in AF occurrence and development, and whether some crucial miRNAs can serve as biomarkers in earlier diagnosis or interventional targets for AF patient in the future.

## Materials and Methods

### Study Population

In Beijing Shijitan Hospital, from Jan. 2011 to Jun 2015, 90 AF patients being prepared for AF radiofrequency ablation operation were classified as paroxysmal AF (ParoAF), persistent (PersAF) or permanent AF (PermAF) groups and were enrolled with a median age of 72.17±4.76 years. The median age of the 90 normal control individuals was 69.40±5.86 years. Every patient had more than five ECGs at different times supporting their diagnosis. Exclusion criteria were age>80 years, hyperthyroidism, uncontrolled hypertension, left ventricular dysfunction with an ejection fraction <40%, severe coronary artery disease, liver or kidney failure, acute or chronic inflammatory disease, and structural heart disease. All patients were receiving regular treatment such as angiotensin-converting enzyme inhibitors, angiotensin receptor blockers, and/or statins, but had to stop anti-arrhythmic drugs at least for 5 days, including β-receptor blockers.

The investigational protocol was approved by the Medical Ethics Committee of Shijitan Hospital of Capital Medical University. Written informed consent was obtained from each participant.

## Methods

### Plasma Collection and Storage

Coronary sinus blood (CSB) and periphery blood (PB) were taken from patients and health subjects (only PB) before and during the AF radiofrequency ablation operation. Whole blood samples (4 mL) were drawn into EDTA-containing tubes and separated into plasma and cellular fractions by centrifugation at 1500 g for 15 minutes. The supernatant was transferred to RNase/DNase-free tubes and stored at -80°C.

### RNA Isolation

Total plasma RNA was harvested with the TRIzol Reagent (Invitrogen Life Technology) and the miRNeasy mini kit (Qiagen) according to the manufacturers’ instructions. In detail, 250 *μ*L EDTA-containing plasma was transferred to an Eppendorf tube and mixed thoroughly with TRIzol Reagent, incubated for 5 minutes at room temperature, and subsequently mixed with 140 *μ*L chloroform. The aqueous phase containing the RNA was carefully removed, and RNA was precipitated by the addition of 100% ethanol. The mixture was applied to an RNeasy mini-spin column and washed several times, and RNA was eluted by the addition of 25 *μ*L RNase-free water. RNA was stored at -80°C until further processing.

### MicroRNA Array and Data Analysis

We performed miRNA expression profiling of the plasma samples from 90 AF patients and 90 healthy donors by using the miRCURY LNA Array (version 18.0) system. RNA samples were labeled with the Exiqon miRCURY Hy3/Hy5 power labeling kit and hybridized on the miRCURY LNA Array (version 18.0) station. Scanning was performed with the Axon GenePix 4000B microarray scanner. GenePix pro V6.0 was used to read image raw intensity. The GEO accession number is GSE76092.

### Real-Time PCR

To confirm the findings obtained by analyzing the miRNA profiles, we measured the expression of dysregulated miRNAs using real-time polymerase chain reaction (PCR). All primers used in this study were synthesized by Bioligo Technologies LTD. (ShangHai, China) and the primers sets specific for each miRNA amplification are shown in Table 1. Before RNA extraction, all serum samples were thawed completely on ice, followed by centrifugation once at 20,000 × g for 15 minutes at 4°C to remove the remaining cell debris. The manufacturers’ protocols were followed by RNA extraction, concentration and quality measurement. The cDNA synthesis kit (Exiqon) was used to make cDNA for miRNA profiling according to the manufacturer’s protocol, using the standard protocol for the ABI PRISM 7000HT Sequence Detection System: 10 s at 95°C and 1 min at 60°C for 40 cycles. Comparison of miRNA expression levels was quantified according to the formula of 2^-ΔΔCT^.

**Table 1:** RT-PCR Primers.

miRNA	Primers
miR-93	5’-GTCGTATCCAGTGCGTGTCGTGGAGTCGGCAATTGCACTGGATACGACCTACCTG-3’
miR-1266	5’-GTCGTATCCAGTGCGTGTCGTGGAGTCGGCAATTGCACTGGATACGACAGCCCT-3’
miR-4279	5’-GTCGTATCCAGTGCGTGTCGTGGAGTCGGCAATTGCACTGGATACGACGAAGCC-3’
miR-3149	5’-GTCGTATCCAGTGCGTGTCGTGGAGTCGGCAATTGCACTGGATACGACATACAC-3’
miR-3171	5’-GTCGTATCCAGTGCGTGTCGTGGAGTCGGCAATTGCACTGGATACGACGATATA-3’
miR-892a	5’-GTCGTATCCAGTGCGTGTCGTGGAGTCGGCAATTGCACTGGATACGACCTACGC-3’

### miRNA Target Prediction

miRNA targets were computationally predicted by using target-prediction programs miRanda, TargetScan and miRBase. The Database for Annotation, Visualization, and Integrated Discovery was used to identify the pathway distribution of the predicted targets. Pathways were presented according to the Kyoto Encyclopedia of Genes and Genomes (KEGG) database, which is a database of biological functional systems, consisting of the component genes and proteins.

### Statistical Analysis

The threshold value for significance used to define upregulation or downregulation of miRNAs was a fold change >1.5, with a value of *P*<0.05 calculated by the *t*-test and variance analysis.

## Results

### Different expression of miRNAs in AF patients and control group

**Fig 1:**
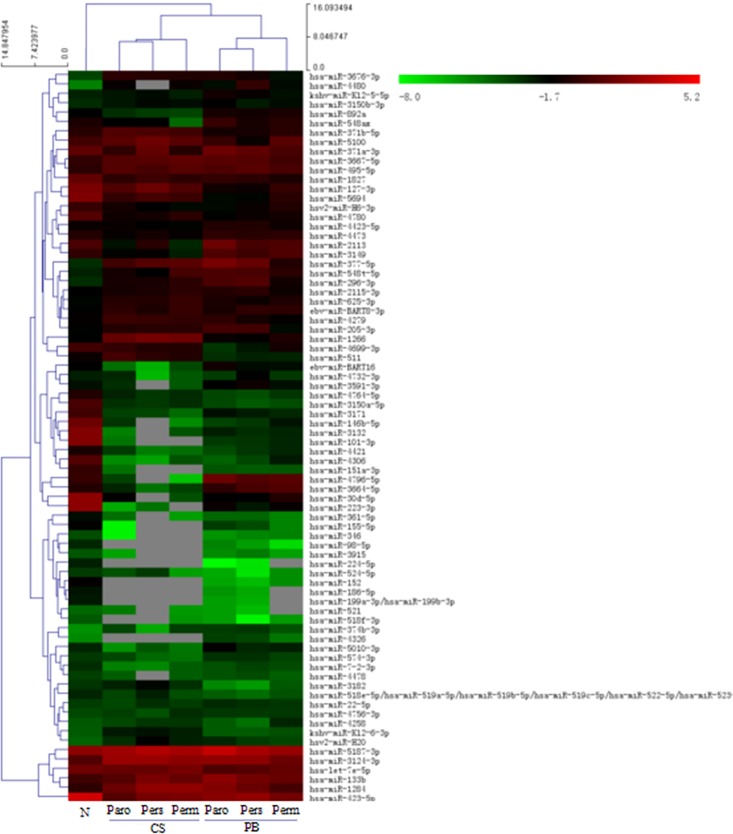
Cluster Analysis of miRNAs Expression in Coronary Sinus Blood and Peripheral Blood of Different Types AF Patients. (Paro: paroxysmal AF; Pers: persistent AF; Perm: permanent AF; N: normal control; CS: coronary sinus; PB: peripheral blood).

196 miRNAs were differentially expressed in patients’ coronary sinus blood (CSB) compared to their peripheral blood (PB); 64 miRNAs had increased levels and 132 miRNAs had decreased expression (Fig 1). Of note, the expression of 8 miRNAs was upregulated significantly (Table 2), including: miR-1266 and miR-4279, increased 11.38- and 2.38-fold, respectively. In contrast, miR-3171 and miR-3664-5p were decreased 0.09- and 0.14-fold, respectively.

**Table 2:** Differential Expression of miRNAs in the CSB vs. PB in the AF Group.

miRNA	CSB expression	PB expression	Fold Change In CSB vs. PB	*P-value*	Expression Tendency
miR-4279	0.8724	0.3673	2.3754	0.0106	up
miR-K12-6-3p	0.2023	0.0716	2.825	0.0011	up
miR-1266	2.4621	0.2163	11.3824	0.0007	up
miR-625-3p	0.6967	0.2729	2.5531	0.0098	up
miR-3664-5p	0.1043	0.726	0.1437	0.0095	down
miR-423-5p	2.532	25.568	0.099	0.0148	down
miR-3591-3p	0.104	0.3201	0.3249	0.0448	down
miR-3171	0.0817	0.9338	0.0875	0.001	down

(CSB: coronary sinus blood; PB: peripheral blood).

In comparison with normal donors’ peripheral blood, 288 miRNAs were differentially expressed in the peripheral blood of AF patients (AF PB); among them, 16 miRNAs were upregulated and 24 miRNAs were downregulated obviously (Fig 1), especially: miR-892a and miR-3149 increased 2.32- and 2.35-fold respectively, while miR-3171 was decreased 0.21-fold (Table 3).

**Table 3:** Differential Expression of miRNAs in the Peripheral Blood of the AF group vs. the Control Group.

miRNA	AF group	Control group	Fold changes in AF G. vs. C.G.	*P-value*	Expression Tendency
miR-892a	0.4823	0.208	2.3182	0.0081	up
miR-3149	1.2278	0.5234	2.346	0.0154	up
kshv-miR-K12-5-5p	0.3451	0.1701	2.0288	0.0427	up
miR-133b	3.0596	1.0599	2.8867	0.0466	up
miR-423-5p	3.9074	25.568	0.1528	0.0182	down
miR-155b	0.0759	0.2402	0.316	0.0355	down
miR-3171	0.1967	0.9338	0.2106	0.0018	down

(AF G.: AF group; C.G.: control group).

### miRNAs expression differences in the ParoAF, PersAF and PermAF groups of patients

**Fig 2:**
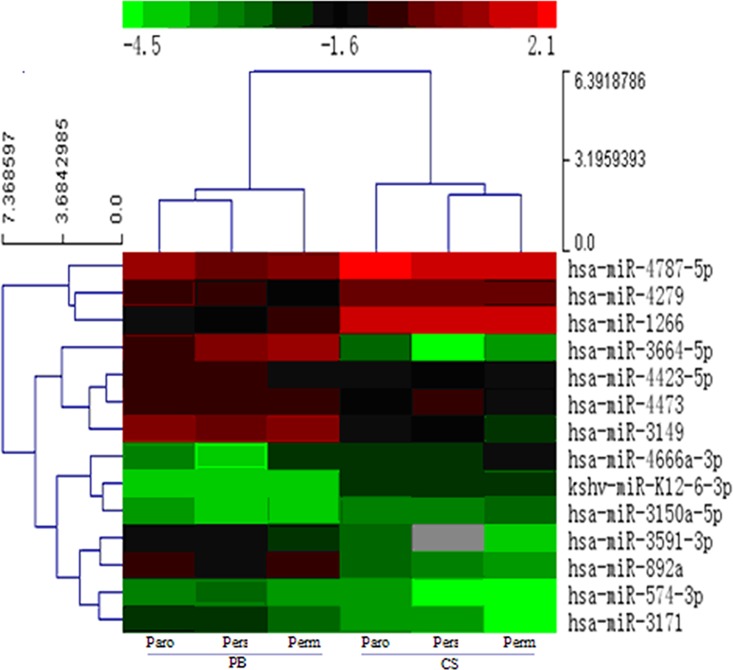
Cluster Analysis of Different Expression of miRNAs in the Coronary Sinus Blood Compared with the Peripheral Blood of AF Patients. (Paro: paroxysmal AF; Pers: persistent AF; Perm: permanent AF; N: normal control; CS: coronary sinus; PB: peripheral blood)

There were 142 miRNAs with differing expression in the CSB compared to the PB of AF patients; among them, 6 miRNAs were upregulated and 8 were downregulated significantly, especially the miR-1266 increased by 5.55-, 8.41- and 4.70-fold, and miR-3171 decreased 0.43-, 0.47- and 0.32-fold in the ParoAF, PersAF and PermAF groups, respectively (Fig 2, Table 4).

**Table 4:** Differential Expression of miRNAs in the Coronary Sinus Blood Compared with the Peripheral Blood of Three Types AF Patients.

miRNA	Paro-AF		Pers-AF		Perm-AF		Exp. Tend.
	F. Change	P	F. Change	P	F. Change	P	
miR-1266	5.5511	0.0146	8.4118	0.0123	4.407	0.0132	up
miR-4279	1.2871	0.0412	1.8665	0.0392	2.2824	0.0387	up
miR-4666a-3p	1.9554	0.0314	4.5005	0.0328	2.1092	0.0314	Up
miR-4787-5p	2.4245	0.0498	2.9854	0.0495	1.9466	0.0478	up
kshv-miR-K12-6-3p	3.0587	0.0179	3.5544	0.0168	2.27	0.0152	up
miR-3150a-3p	1.4842	0.0095	1.7927	0.0099	1.7651	0.01	Up
miR-3664-5p	0.229	0.0495	0.0366	0.0484	0.0721	0.0499	down
miR-3591-3p	0.4545	0.0064	1.0043	0.0499	0.2846	0.0134	down
miR-892a	0.2927	0.0081	0.2534	0.0094	0.1793	0.009	down
miR-4423-5p	0.5553	0.0442	0.5505	0.0399	0.7136	0.0417	down
miR-4473	0.5009	0.035	0.7234	0.0319	0.5159	0.0378	down
miR-574-3p	0.6909	0.0469	0.3726	0.0451	0.4365	0.0431	down
miR-3149	0.2191	0.0256	0.3432	0.0248	0.1356	0.0231	down
miR-3171	0.4294	0.0079	0.4723	0.008	0.3211	0.0085	down

{Paro: paroxysmal AF; Pers: persistent AF; Perm: permanent AF; F. Change: fold change; Exp. Tend.: expression Tendency}.

**Fig 3:**
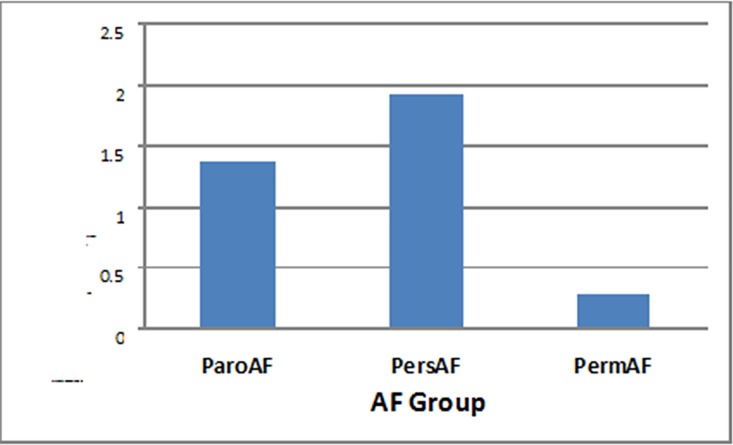
The differential expression of miRNAs among ParoAF, PersAF and PermAF groups of AF patients. (ParoAF: paroxysmal AF; PersAF: persistent AF; Perm: permanent AF).

The differential expression of miRNAs among ParoAF, PersAF and PermAF groups of AF patients with variance analysis was shown that the ParoAF compared with PersAF group there was no significance (F = 1.478, P = 0.235), but dramatically difference in ParoAF and PersAF groups compared with PermAF group respectively (F = 16.877, P = 0.000; F = 16.628, P = 0.000; Table 4, Fig 3).

### Real-Time PCR Validation of the Profiling Data

**Fig 4:**
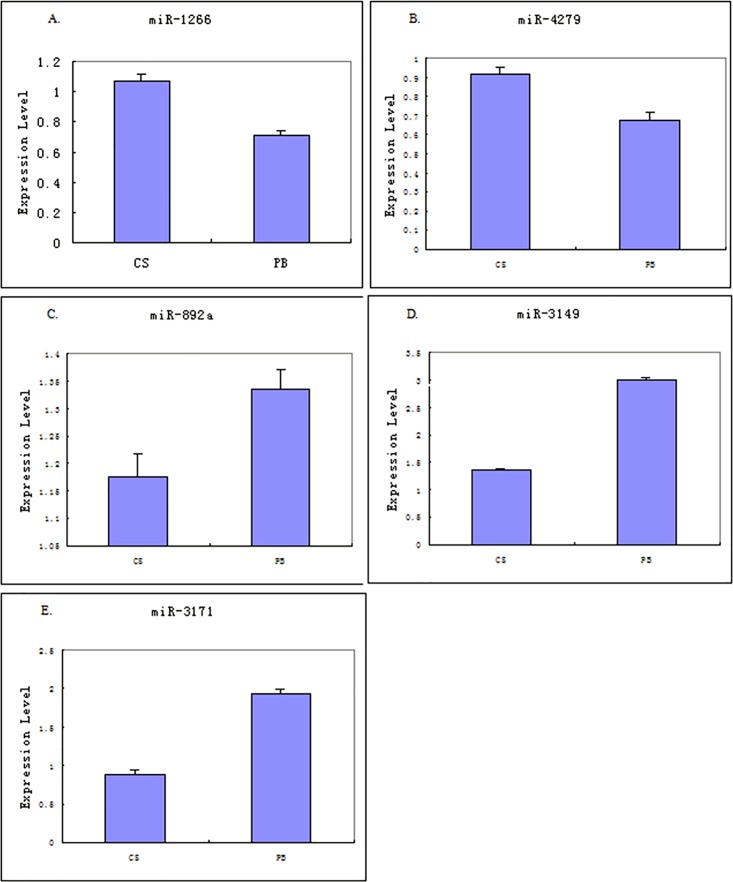
Real-Time PCR Validation of the Profiling Data. (A. miR-1266; B. miR-4279; C. miR-892a; D. miR-3149; E. miR-3171; CS: coronary sinus; PB: peripheral blood).

The chip data of five candidate microRNAs (miR-1266, miR-4279, miR-892a, miR-3149, and miR-3171) were validated by using real-time PCR. The expression levels of these five miRNAs confirmed the previously observed significant upregulation or downregulation (Fig 4).

### miRNA Targets Prediction Results

Key miRNA targets are shown in Table 5, which was predicted by using the miRBase, miRanda, and TargetScan databases.

**Table 5:** miRNA Targets Prediction Results.

miRNA	target genes regulating ion channel proteins
has-miR-4279	KCNH2, KCNE1, KCND1, KCNC4, KCNN3
has-miR-892a	RYR2, KCNH2, KCND2, GJA1, KCNQ5, KCNA2, KCNA4
has-miR-3149	GJA1, HCN1, KCND2, KCNQ5, KCNC4, KCNN2, KCNE3, KCNA1, KCNA3, KCNA4
has-miR-3171	GJA5, RYR2, KCNC4, KCNA4, KCNC2, SCN5A, CAMK2N1, CACNB2, CACNB3, CACNB4
has-miR-4666a-3p	KCNE4, KCNQ3, KCNC1, KCNG3, KCNJ3, HCN4, CACNA1C, CAMSAP1, CAMTA1
has-miR-1266	SCN5A, KCNH2, KCNE1, KCNA6, KCNA7, KCNC3, KCNB1, KCNG4, KCNH5, KCNH8, KCNP4, KCNJ1, KCNJ11, KCNJ3, KCNJ5, KCNK1, KCNK10, KCNMA1, KCNS1, KCNS2, KCNU1

## Discussion

In our study, differential expression patterns of miRNAs were evaluated in the coronary circulation and peripheral plasma of 90 patients with paroxysmal, persistent, or permanent AF. Through the comparison of these miRNA expression patterns in the CSB and PB of patients, and with control PB, we tried to discover the potential value of coronary circulating miRNAs expression and regulation in patients with AF and to further explore the key miRNAs which may take part in the occurrence and propagation processes of AF and provide evidence for earlier warning and intervention of the disease.

### The significance of miRNAs in coronary sinus blood compared to peripheral blood

In our study, there were 142 miRNAs differentially expressed in the comparison of CSB and PB in the AF groups. Among them, 6 miRNAs were upregulated significantly, and 8 were downregulated (Table 2 and Fig 2). The differential expression of miRNAs in the CSB compared with the autologous PB of patients (Table 2) and the CSB compared with the PB of healthy controls (Table 3) was obviously shown. Zhang Y., *et al*. [24] has previously reported differential expression of miRNAs in the peripheral blood of paroxysmal and persistent AF patients by using a chip test. miR-3169, miR-3612, miR-634, miR-376a, and miR-377 were upregulated more than twofold; meanwhile, 3 miRNAs were down regulated, including: kshv-miR-K12-5, miR-378c and miR-204. Circulating miRNAs in AF patients were sequenced by Liu, *et al*. [25], and the expression levels of miR-146a, miR-150 and miR-19a were found to be decreased; notably, they concluded that a reduction in circulating miR-150 was significantly associated with AF. Additionally, they found that plasma levels of CRP were negatively correlated with the plasma levels of miR-150. These findings are not consistent with ours. There might be several explanations: both studies had small sample sizes, and they did not include permanent AF patients. Other reasons may include: different severity and stages of the disease and different versions of the detection chips and detective methods; moreover, the high sensitivity of high throughput detection may cause the observed deviation in results. Furthermore, to some extent, crucial miRNAs that were detected in our study are newly discovered, which may be related to the use of coronary sinus blood and the latest chip version, which we employed.

### The differential expression of miRNAs among ParoAF, PersAF and PermAF groups of AF patients

The differential expression of miRNAs in three types of AF patients was shown in Table 4. With variance analysis, we found out that there was obviously significance among ParoAF and PersAF groups compared with PermAF group respectively (Fig 3), and indicated that the difference manifestation of different kinds of AF is not only in clinic, but also in miRNA regulation mechanism. The more different phase of AF in clinic was, the much different regulation in miRNAs may be. According to the mechanism and the targets of these miRNAs, the difference is not only involved in electrical remodeling, but also in constructional remodeling. Even if there was no significant in ParoAF and PersAF groups in statistics, some miRNAs has already changed much differently, such as miR-1266, miR-3664-5p and miR-4666a-3p, etc. (Table 4). So, the levels of miRNA changes may reflect the severity of AF clinical and pathophysiological advance. Also, combining with our previous study, the AF radiofrequency ablation can reverse the circulating miRNA changes [23], further indicated that the importance of miRNA regulation in AF occurrence and development. Anyway, our results still remain to be further verified, and more studies need to further reveal the values of differential expression of miRNAs in 3 types of AF patients.

### Meaning of newly discovered miRNAs

#### miRNAs regulating ion channel genes

Compared with the autologous PB, the expression levels of miR-1266, miR-4279 and miR-4666a-3p were all upregulated significantly in the CSB. According to target gene prediction, various ion channel genes are regulated by those miRNAs. Characterization of the Na^+^ leak channel (NALCN), which was regulated by miR-1266, has been linked to a role in conducting the background Na^+^ current that depolarizes the resting membrane of the pacemaker and regulates cell excitability [26]. Arrhythmias occurred in rats lacked the CACNA1E gene that is targeted by miR-1266 [27]. The Ca^2+^ channel gene (CACNA1C), as well as the Ca^2+^-activated K^+^ channel (KCNN3) is repressed by miR-4279. It has been confirmed that upregulation of KCNN3 can increase the risk of AF in an evidence-based medicine trial [28]. Transgenic animal experiments by Yang. B, *et al*. [29] confirmed that CACNA1C participates in the regulation of AF. Large scale genetic screening had verified that KCNE1, KCNH2 and KCNJ5 could be regulated by miR-1266 and miR-4279, and they both are related to the development of AF [30–34]. CACNA1C and KCNG3 are affected by miR-4666a-3p, and these two genes also play important part in regulating neurotransmitter release, neuronal excitability, heart rate, smooth muscle contraction and so forth.

#### miRNAs involving in constructional remodeling

The critical miRNAs which regulate AF may not only involve ion channels but also be related to a variety of biological processes such as cell proliferation, senescence, apoptosis, inflammation and fibrosis. It has been reported that miR-1266 plays an important role in the regulation of the inflammatory response [35]. miR-892a was identified as a negative regulator of CYP1A1 expression which is a member of the cytochrome P450 enzyme family [36]. Further studies are needed to reveal the relationship between these key miRNAs we reported here and AF.

### miRNAs secreted by myocardium

#### Myocardial miRNAs

Those miRNAs were significantly increased in the CSB compared with the PB of AF patients, indicated that they may be secreted by the myocardium, specifically or in greater amounts in atrial myocardium as in atrial fibrillation pathophysiological conditions. Metabolic and regulating characteristics of the above myocardial miRNAs, such as miR-1266, miR-4279 and miR-4666a-3p, supported that they may be the crucial factors in AF regulation. Further research is warranted to confirm their potentials as interventional targets.

#### Non myocardial miRNAs

Compared to the peripheral blood of normal subjects, the levels of miR-3171 were lower in the CSB and PB of AF patients, indicating that it is mainly secreted by tissues other than the myocardium, and it may participate in the regulation of AF through coronary circulation. The continuous decrease of miR-3171 in patients with paroxysmal, persistent and permanent AF makes it possible for it to become a biomarker of this disease.

The expression of miR-892a and miR-3149 were higher in the PB group of patients than the CSB group of patients as well as the normal control group. There was no difference between the CSB group and the normal control group. This indicates that they were produced by tissues other than cardiac cells, and act as the regulators in the coronary circulation. Although both of them were reduced in the CSB of patients but increased in the PB of patients, they are likely to become diagnostic markers of AF. Additionally, it needs to be further confirmed that whether many factors such as aging, hypertension, hyperlipidemia and other systemic diseases or states of sympathetic activation and/or inflammation may affect the result.

## Conclusion

The differential expression of miRNAs in coronary circulation in Three Types AF Patients in our study indicated that: 1) the expression of miRNAs in the CSB may better reflect the real metabolism, expression and regulation status of miRNAs in patients with AF; 2) the levels of miRNA changes may reflect the severity of AF clinical and pathophysiological advance; and 3) the miR-3171, miR-892a and miR-3149, variably expressed from the early to the end stage of AF in PB, may be used as biomarkers for earlier diagnosis of AF; the miR-1266, miR-4279, and miR-4666a-3p, obviously increased in the CSB of AF patients, may serve as potential intervening targets for AF in the future.
